# Detection of opportunistic bacterial pathogens with intrinsic amoxicillin- and cephalosporin-resistance in wild koala faecal microbiomes

**DOI:** 10.1099/mic.0.001773

**Published:** 2026-09-09

**Authors:** Fiona K. McDougall, Kalani Paranagama, Wayne S.J. Boardman, Kelly Wyres, Michelle L. Power

**Affiliations:** 1 School of Natural Sciences, Faculty of Science and Engineering, Macquarie University, Sydney, NSW 2109, Australia; 2 Department of Infectious Diseases, School of Translational Medicine, Monash University, 85 Commercial Rd, Melbourne, Victoria, 3004, Australia; 3 Centre to Impact AMR, Monash University, Clayton, Victoria, 3800, Australia; 4 School of Animal and Veterinary Sciences, College of Sciences, Adelaide University, Adelaide, SA 5371, Australia; 5 Department of Infection Biology, Faculty of Infectious and Tropical Diseases, LondonSchool of Hygiene & Tropical Medicine, London, UK

**Keywords:** antimicrobial stewardship, *Citrobacter*, *Klebsiella*, One Health, *Phascolarctos cinereus*, *Scandinavium*

## Abstract

Opportunistic bacterial pathogens frequently associated with human clinical infections, including antimicrobial-resistant strains, are infiltrating the microbiomes of wild animals, where they have the potential to negatively impact wildlife health. Bacterial genes conferring resistance to amoxicillin have previously been reported in koala (*Phascolarctos cinereus*) faecal DNA. Koalas are facing several key threats, including wildfires, and affected individuals may receive amoxicillin therapy to treat burn wounds. This study aimed to identify the species of amoxicillin-resistant bacteria in koala gut microbiomes and determine if they are opportunistic pathogens. Faecal samples collected from 98 wild-caught koalas were cultured using amoxicillin-supplemented media to isolate amoxicillin-resistant Gram-negative enteric bacteria. Isolates were screened using 16S rRNA PCR and Sanger sequencing to identify opportunistic pathogenic species, which then underwent whole-genome sequencing and antimicrobial susceptibility testing. Intrinsically amoxicillin-resistant opportunistic pathogens were obtained from 9.2% (9/98) of koala faecal samples and comprised *Klebsiella oxytoca* (6/98, 6.1%), *Klebsiella pneumoniae* (1/98, 1.0%) and *Citrobacter* spp. (2/98, 2.0%). Seven of nine amoxicillin-resistant opportunistic pathogens also exhibited cephalosporin resistance. Four *K. oxytoca* isolates belonged to lineages associated with human clinical infections, which also have the potential to cause disease in koalas, including fatal systemic infections in pouch young. The presence of amoxicillin- and cephalosporin-resistant strains may also increase the risk of gut dysbiosis and opportunistic infections when penicillins or cephalosporins are required to treat bacterial infections in koalas, highlighting the importance of good antimicrobial stewardship. The study findings demonstrate the One Health perspective of microbial pathogens and the intertwined microbial ecology between humans and wildlife.

## Data Summary

All whole-genome sequencing raw data have been uploaded to the NCBI Sequence Read Archive (SRA) database under BioProject PRJNA1241462 (https://www.ncbi.nlm.nih.gov/bioproject/PRJNA1241462) and BioSamples SAMN47556974 to SAMN47556984. Assembled genomes are available for *Klebsiella oxytoca* at PubMLST (https://pubmlst.org/organisms/klebsiella-oxytoca), *Klebsiella pneumoniae* at Bacterial Isolate Genome Sequence Database (BIGSdb, https://bigsdb.pasteur.fr/klebsiella/) and *Citrobacter* spp. at PubMLST (https://pubmlst.org/organisms/citrobacter-spp). Individual isolate BIGSdb and NCBI SRA and assembly accession numbers are provided in Table S2.

## Introduction

Opportunistic bacterial pathogens frequently associated with clinical infections in humans have infiltrated the microbiomes of many wild animal species, with many of these pathogens also exhibiting resistance to antibiotics [1, 2]. These bacterial pathogens have the potential to cause negative impacts for the microbiome and health status of wildlife and pose a conservation threat to endangered species [3, 4]. Opportunistic pathogens have been responsible for causing disease in wild animals – for example, hypervirulent *Klebsiella pneumoniae* infection in sea mammals (sea lions, seals and otters), which frequently leads to fatal pneumonia [5–7], and methicillin-resistant *Staphylococcus aureus* skin infections in European hedgehogs (*Erinaceus europaeus*) [8–10]. Opportunistic bacterial pathogens exhibiting antimicrobial resistance (AMR) now impact human, domestic animal, wildlife and environmental health; thus, a One Health approach to combating AMR is critically important [11].

In Australian wildlife, opportunistic pathogenic strains of *Escherichia coli* and *K. pneumoniae* known to cause clinical infections in humans have been reported in several wildlife species, including fruit bats (grey-headed flying fox; *Pteropus poliocephalus*) [12–14] and silver gulls (*Chroicocephalus novaehollandiae*) [15, 16]. Many of these bacterial pathogens also exhibited resistance to antibiotics deemed medically important by the World Health Organization [17], including fluoroquinolones, beta-lactams (inclusive of penicillins, cephalosporins and carbapenems), trimethoprim plus sulfonamides and tetracyclines [12–16].

The beta-lactam group of antibiotics, particularly penicillins and cephalosporins, are the most frequently administered antibiotics to humans [18, 19], companion animals and livestock [20], and also have considerable use in wildlife medicine [21]. In Gram-negative bacteria, resistance to beta-lactam antibiotics is typically conferred by genes encoding beta-lactamases, which includes *bla*TEM, *bla*OXA, *bla*SHV, *bla*CTX-M, *bla*KPC, *bla*NDM, *bla*IMP and *ampC* enzymes (includes *bla*CMY) [22]. Some species of Gram-negative bacteria are intrinsically resistant to beta-lactams, typically penicillins and sometimes cephalosporins, as they carry chromosomal beta-lactamase genes such as *bla*SHV in *K. pneumoniae*, *bla*OXY in *Klebsiella oxytoca*, *bla*ADC in *Acinetobacter baumannii* and *bla*CMY in some *Citrobacter* spp. [22, 23]. Many strains of Gram-negative bacteria have acquired beta-lactamase genes, usually via transfer of plasmids between bacterial strains, with most plasmid-associated beta-lactamase genes having an intrinsic chromosomal origin [22]. Of the acquired beta-lactamases, *bla*TEM (confers resistance to ampicillin, amoxicillin and sometimes cephalosporins) is one of the most common found in Gram-negative bacteria [23]. In a previous study of the koala (*Phascolarctos cinereus*), the beta-lactamase gene *bla*TEM was identified in the faecal DNA of 2.6% of koalas [24]. However, the bacterial species carrying the *bla*TEM genes are unknown, as is the pathogenicity of these strains [24]. It is also unknown if other common beta-lactamase-resistance genes, such as *bla*OXA, *bla*SHV, *bla*CTX-M and *bla*CMY [22], are present in koala faecal DNA.

During the catastrophic wildfires in the Australian summer of 2019–2020, hundreds of fire-affected koalas were treated for burn wounds and other injuries or disease at wildlife hospitals [25–27]. Analysis of koala veterinary treatment records determined the most commonly administered antibiotic class was penicillins (119/355, 33.5% of admitted koalas), of which amoxicillin was the most frequently used (94/355, 26.5% of admitted koalas) [28]. The majority of these administered penicillins (95/119, 79.8%) were for prophylactic treatment of non-infected burn wounds [28].

Antibiotic administration has the potential to select for resistant opportunistic pathogens that are already present in the animal’s microbiome, providing a selective advantage allowing these strains to establish infections [29, 30]. In koalas, the risk of antibiotic-induced fatal gastrointestinal dysbiosis caused by the inadvertent loss of specialized gut bacteria essential for digesting eucalypt leaves [31, 32] may be exacerbated by the presence of antibiotic-resistant opportunistic pathogens [33]. Common Gram-negative opportunistic pathogens that colonize the gut microbiome include *E. coli*, *K. pneumoniae, Enterobacter cloacae* and *Citrobacter freundii* [34–36], of which *E. coli*, *K. pneumoniae* and *E. cloacae* have been reported in the koala gut microbiome [37, 38]. These opportunistic pathogens can extend from the gut microbiome and cause extraintestinal infections, including pneumonia, sepsis, meningitis, bloodstream, wound and urinary tract infections [29]. Their pathogenicity is typically associated with the carriage of numerous virulence factors and clinical strains are often multidrug resistance (MDR) [34–36].

This study aimed to identify the bacterial species of amoxicillin-resistant Gram-negative isolates detected in the gut microbiome of wild koalas and to determine if they are opportunistic pathogens. Detected bacterial pathogens underwent genetic characterization to determine if they are strains known to cause clinical disease in humans and other animals and to identify the genes responsible for conferring AMR. Bacterial isolates also underwent antimicrobial susceptibility testing (AST) against a panel of antibiotics to identify resistance to additional classes of antibiotics. Understanding the dynamics of resistant opportunistic pathogens in the koala gut microbiome will contribute to identifying potential disease threats and risk factors associated with poor outcomes when antibiotics are required for treating bacterial infections in koalas.

## Methods

### Koala faecal sampling

Faecal swab samples (*n*=98) were opportunistically collected from wild-caught koalas in October 2022 during a previous study (Table S1, available in the online Supplementary Material) [24]. All koalas (*n*=98) were captured in Belair National Park (BNP), which is located directly adjacent to the eastern border of the city of Adelaide, South Australia and extends into the Mount Lofty Ranges. Faecal swab samples were obtained by rectal swab using the FecalSwab^TM^ system (COPAN, Brescia, Italy) and stored at 4 ˚C until transferred to the laboratory. Aliquots of koala FecalSwab media were stored in 35% glycerol and frozen at −80 ˚C until selective culture was performed. In the previous study, faecal DNA samples (*n*=98) extracted from faecal pellets collected from the same koalas used in this study were screened for *bla*TEM genes using PCR; however, none of the 98 faecal DNA samples were *bla*TEM-positive [24]. These faecal DNA samples were not screened for any additional beta-lactam-resistance genes. Metadata was provided for all individual koalas, including age, body weight, sex and body condition score (BCS) (emaciated, poor, fair, good or excellent) (Table S1).

### Selective culture for amoxicillin-resistant enteric bacteria

Koala faecal samples were cultured using media supplemented with 10 mg l^−1^ amoxicillin (Sigma, St. Louis, MO, USA) to detect beta-lactam-resistant Gram-negative enteric bacteria, based on the Enterobacterales European Committee on Antimicrobial Susceptibility Testing (EUCAST) clinical breakpoints (v 14.0) for amoxicillin (available at https://www.eucast.org/bacteria/document-archive/). Faecal swab media plus 35% glycerol (0.3 ml) was inoculated into 5 ml of MacConkey broth (Oxoid, Basingstoke, UK) supplemented with 10 mg l^−1^ amoxicillin (Sigma, St. Louis, MO, USA) and incubated at 37 °C overnight. MacConkey broth cultures were subsequently inoculated onto MacConkey agar (Oxoid, Basingstoke, UK) supplemented with 10 mg l^−1^ amoxicillin (Sigma, St. Louis, MO, USA) and incubated at 37 °C overnight. Agar plates were examined for colony growth and positive plates were sub-cultured to acquire pure bacterial isolates. To obtain DNA from koala isolates, one colony was inoculated into 10 ml Luria–Bertani broth (Difco Laboratories, Detroit, MI, USA) and genomic DNA was extracted using the ISOLATE II Genomic DNA Kit (Bioline, London, UK).

### Species identification of amoxicillin-resistant bacterial isolates

To identify the bacterial species of amoxicillin-resistant isolates obtained from koalas, a 16S rRNA PCR was performed using the universal eubacterial primers f27 and r1492 [39], as previously described [40]. The 16S rRNA PCR products were sequenced using Big Dye Terminator version 3.1 and ABI 3730 Capillary Sequencers (Applied Biosystems, Foster City, CA, USA) at The Ramaciotti Centre for Genomics (University of New South Wales, Sydney, Australia). Resulting sequences were analysed using Geneious Prime software version 2023.1 (Biomatters Limited, Auckland, New Zealand) with preliminary bacterial species determined using blastn searches (https://blast.ncbi.nlm.nih.gov/Blast.cgi). Bacterial species identified as opportunistic pathogens by 16S rRNA sequencing were selected to undergo further analysis using whole-genome sequencing (WGS) and AST.

### WGS of amoxicillin-resistant isolates

Genomic DNA concentrations were determined using a Qubit dsDNA HS Assay kit (Invitrogen, Waltham, MA, USA). WGS was performed with paired-end (2×150 bp) sequencing runs using the Illumina NextSeq 1000 system at the Ramaciotti Centre for Genomics (Sydney, NSW, Australia). Raw sequence reads were assembled as *de novo* genome sequences using Velvet short read assembler version 1.2 [41] in Geneious Prime software versions 2023.1 to 2024.0 (Biomatters Limited, Auckland, New Zealand). Raw sequence reads for all isolates were uploaded to the NCBI Sequence Read Archive (SRA) under BioProject PRJNA1241462 – BioSample IDs SAMN47556974 – SAMN47556996 (available at https://www.ncbi.nlm.nih.gov/bioproject/PRJNA1241462).

All *K. oxytoca* assemblies were uploaded to the PubMLST *K. oxytoca* species complex isolates database – IDs 1848 to 1853 (available at https://pubmlst.org/organisms/klebsiella-oxytoca). The *K. pneumoniae* assembly was uploaded to the *K. pneumoniae* species complex database – ID 120483 (available at https://bigsdb.pasteur.fr/klebsiella/). *Citrobacter* spp. assemblies were uploaded to the PubMLST *Citrobacter* spp. isolates database – IDs 1067 and 1068 (available at https://pubmlst.org/organisms/citrobacter-spp). Individual isolate PubMLST, Bacterial Isolate Genome Sequence Database (BIGSdb) and NCBI SRA and assembly accession numbers are provided in Table S2.

### Bacterial strain typing using WGS data

For isolates for which the bacterial species could not be confirmed using 16S rRNA sequencing alone, WGS and ribosomal multilocus sequence typing (rMLST) were used to identify species (available at https://pubmlst.org/species-id) [42]. For presumptive *K. oxytoca* isolates, the PubMLST *K. oxytoca* species complex database (available at https://pubmlst.org/organisms/klebsiella-oxytoca) was used to confirm species and assign isolates to a sequence type (ST) [43]. For presumptive *K. pneumoniae*, the BIGSdb-Pasteur *K. pneumoniae* species complex database (https://bigsdb.pasteur.fr/cgi-bin/bigsdb/bigsdb.pl?db=pubmlst_klebsiella_isolates) was used to confirm species and assign a ST [44, 45], while the O type and K locus were determined using Kaptive v3.1.0 via Pathogenwatch [46, 47]. For presumptive *Citrobacter* spp. isolates, the PubMLST *Citrobacter* spp. isolates database (available at https://pubmlst.org/organisms/citrobacter-spp) was used to confirm species and assign a ST [43].

### Detection of antibiotic resistance genes, virulence factors and plasmids

Genome assemblies for all isolates were screened for antimicrobial resistance genes (ARG) using ResFinder Version 4.7.2 (available at https://genepi.food.dtu.dk/resfinder) [48, 49]. The *Klebsiella* isolates were screened for ARGs and virulence factors (aerobactin, colibactin, salmochelin, yersiniabactin and *rmpADC*) using the Kleborate galaxy plugin (Galaxy Version 2.3.2+galaxy1) (available at https://usegalaxy.eu/?tool_id=kleborate) [44, 50]. Isolate assemblies were screened for plasmids using PlasmidFinder 2.1, with search parameters set at 80% for identity, 60% minimum length and *Enterobacteriaceae* [51] (available at https://www.genomicepidemiology.org/services/).

### Antimicrobial susceptibility testing

All amoxicillin-resistant isolates underwent further AST against a panel of 11 antibiotics: ampicillin, amoxicillin+clavulanic acid, cefazolin, cefotaxime, imipenem, ciprofloxacin, gentamicin, trimethoprim, trimethoprim+sulfamethoxazole, chloramphenicol and tetracycline (Table S3). AST was performed using the disc diffusion method established by the EUCAST [52] with Mueller Hinton agar (Oxoid, Basingstoke, UK) and Oxoid antibiotic discs (Oxoid, Basingstoke, UK) (Table S3). A negative control isolate (*E. coli* FF1170 – Enterobase Barcode ESC_JA9915AA) was included in all disc diffusion AST of koala isolates, as previously described [12]. Isolates were evaluated as susceptible or resistant using EUCAST clinical breakpoints (v 14.0) available at https://www.eucast.org/bacteria/document-archive (Table S3). Where EUCAST breakpoints were unavailable, susceptibility was determined using the Clinical and Laboratory Standards Institute (CLSI) breakpoint criteria (CLSI M100 ED34 : 2024) available at https://em100.edaptivedocs.net/BulletinBoard.aspx#Document+Updates (Table S3). MDR is defined as having acquired phenotypic resistance to at least one antibiotic in at least three categories of antimicrobials [53].

### Statistical analysis

To determine if there were associations between carriage of opportunistic pathogenic bacteria and host metadata traits, Fisher’s exact test was used in R (4.5.1) (https://CRAN.R-project.org/package=epiR) and RStudio (2025.05.1+513) (https://posit.co/download/rstudio-desktop/). A *P*-value<0.05 was considered statistically significant.

### Phylogenetic analysis and genetic relationships of koala isolates

To visualize the Australian koala *Klebsiella* isolates within the global context, additional *K. oxytoca* of the same ST were identified in Pathogenwatch (available at https://next-docs.pathogen.watch). To provide broader context against Australian human clinical isolates, all human clinical *K. oxytoca* isolates from Stewart *et al*. [54] were added to the collection. For *K. pneumoniae*, the koala isolate was assigned to a Life Identification Number (LIN) Sublineage (stable groupings that reflect deep-branching phylogenetic lineages and are similar to seven-gene MLST derived clonal groups) [55], and isolates of the same LIN Sublineage were identified on the BIGSdb (https://bigsdb.pasteur.fr/cgi-bin/bigsdb/bigsdb.pl?db=pubmlst_*klebsiella*_isolates). A total of 70 *K*. *oxytoca* isolates (koala *n*=6, human *n*=63 and environmental *n*=1) and 14 *K*. *pneumoniae* isolates (koala *n*=1, human *n*=8, domestic animals *n*=2, environmental *n*=1 and unknown *n*=2) were used for phylogenetic analysis.

For *K. pneumoniae* and *K. oxytoca* isolates, single nucleotide variants (SNVs) were identified by mapping reads against reference genomes; NTUH-K2044 (NCBI accession: NC_012731.1) for *K. pneumoniae* and the Kox100 complete genome (NBCI accession: CP089411.1) for *K. oxytoca*, with RedDog (https://github.com/katholt/reddog-nf). Using the resulting alignments, core-genome maximum-likelihood phylogenies (where core genes are defined as those that were annotated in the reference genome and present, with coverage ≥95% and mean read depth ≥5×, in all isolates) were inferred for *n*=14 *K*. *pneumoniae* and *n*=70 *K*. *oxytoca* isolates, respectively, using FastTree v2.1.0. Pairwise SNV distances were calculated using the Reddog SNV matrix, which was analysed to identify the closest isolate to the Australian koala isolates within each of the isolate collections.

Unlike for *Klebsiella* spp., *Citrobacter* spp. are not currently represented in Pathogenwatch. Therefore, to identify publicly available genomes that were closely related to those sequenced here, all *Citrobacter* isolates available in the PubMLST *Citrobacter* spp. genome collection (available at https://pubmlst.org/organisms/citrobacter-spp) were used (*n*=447). The genetic relationships of koala and PubMLST *Citrobacter* isolates were determined in PubMLST using GrapeTree and MSTree V2 [56] to construct a minimum spanning tree based on the PubMLST *Citrobacter* multilocus sequence typing (MLST) scheme (includes seven MLST housekeeping loci) and the ribosomal MLST scheme (includes 53 ribosomal loci) [43]. A refined GrapeTree using all *Citrobacter braakii* isolates available in the PubMLST *Citrobacter* genome collection (*n*=54) was also constructed, as described above [56]. Branch lengths indicate the allelic differences between isolates.

*Scandinavium* spp. are not represented in Pathogenwatch, nor is there a *Scandinavium* PubMLST database. Therefore, we searched NCBI for publicly available *Scandinavium spp* genomes. A *Scandinavium* spp. whole-genome SNV tree comprised of the two koala *Scandinavium* spp. isolates, two representative *Klebsiella aerogenes* isolates and *Scandinavium* spp. assembled genomes available in NCBI (*n*=22) (available at https://www.ncbi.nlm.nih.gov/datasets/genome/). The midpoint-rooted core genome SNV tree was constructed using CSI Phylogeny 1.4 (available at https://www.genomicepidemiology.org/services/) [57], with the reference genome *Scandinavium goeteborgense* strain CCUG 66741 (NCBI Assembly ASM393589v2).

Average Nucleotide Identities (ANIs) were calculated using FastANI v1.3.4 (https://github.com/ParBLiSS/FastANI), with default parameters.

## Results

### Amoxicillin-resistant Gram-negative enteric bacteria detected in koalas

Amoxicillin-resistant bacterial growth was observed in 17 of 98 (17.3%) koala faecal samples on MacConkey agar supplemented with 10 mg l^−1^ amoxicillin, and pure bacterial isolates were obtained for all 17 positive samples. DNA sequencing of 16S rRNA PCR products predicted 11 of 17 isolates as opportunistic bacterial pathogenic species and the remaining six isolates as rare pathogenic bacterial species (Table 1). Presumptive opportunistic pathogens comprised *K. oxytoca* (*n*=6, 6.1%), *K. pneumoniae* (*n*=1, 1.0%), *Citrobacter* spp. (*n*=2, 2.0%), and two isolates could not be differentiated between *K. aerogenes and Scandinavium* spp. (Table 1). The 11 isolates predicted to be opportunistic pathogens were selected to undergo WGS and AST, and the six isolates identified as rare pathogenic bacterial species were not analysed further (Table 1).

**Table 1.** Bacterial species identification (using 16S rRNA PCR) and predicted pathogenicity of 17 amoxicillin-resistant bacterial isolates cultured from 100 faecal samples obtained from wild-caught koalas in BNP, South Australia

Bacterial species – 16S rRNA PCR – closest blastn match	Closest NCBI accession no. (% identity to closest blastn match)	No. of isolates (% koalas)	Predicted pathogenicity to humans and other mammals	Selected for WGS and AST
*K. oxytoca*	CP188760 (98.6–99.7%)	6 (6.1%)	Opportunistic pathogen	Yes
	MT453831 (99.9%)			
	CP089411 (98.5%)			
	CP196091 (100%)			
*K. pneumoniae*	MK386773 (99.3%)	1 (1.0%)	Opportunistic pathogen	Yes
*Citrobacter* spp.	KR189801 (100%)	2 (2.0%)	Opportunistic pathogen	Yes
	MH191146 (100%)			
*K. aerogenes*/*Scandinavium* spp.	MT827114 (98.7–98.8%)/PQ325750 (98.9%)	2 (2.0%)	Opportunistic pathogen (*K. aerogenes*)/Rare pathogen (*S. goeteborgense*)	Yes
*Erwiniaceae*	PQ483424 (95.5%) OR910566 (99.9%)	2 (2.0%)	Rare pathogen (Plant pathogen)	No
*Hafnia* spp.	PP663890 (100%) CP139992 (100%)	2 (2.0%)	Rare pathogen (Gut commensal/Environmental)	No
*Serratia* spp.	KR150404 (100%) OP999366 (100%)	2 (2.0%)	Rare pathogen (Environmental)	No

### WGS of koala amoxicillin-resistant bacteria predicted to be opportunistic pathogens

WGS and genomic characterization confirmed that 9 of 11 bacterial isolates that underwent WGS were species considered opportunistic pathogens – 7 *Klebsiella* spp. and 2 *Citrobacter* spp. The *Klebsiella* isolates were identified as *K. oxytoca* (*n*=6) and *K. pneumoniae* (*n*=1), and the *Citrobacter* isolates were *C. braakii* and an undefined *Citrobacter* species (Table 2). Both *Citrobacter* isolates, one *K. oxytoca* isolate and the *K. pneumoniae* isolate were assigned novel STs and five *K. oxytoca* were assigned to previously reported STs (Table 2). For the two isolates that could not be assigned to a bacterial species using 16S rRNA PCR sequence analysis, rMLST identified both isolates as *Scandinavium* spp., with the closest match being *Scandinavium lactucae* (Table 2)*. Scandinavium* spp. are considered rare pathogens rather than opportunistic pathogens; thus, overall 9 of 98 (9.2%) koala faecal samples harboured opportunistic bacterial pathogens.

**Table 2.** Genomic characterization, antibiotic resistance profiles and predicted pathogenicity of 11 intrinsically amoxicillin-resistant bacterial isolates from koala faecal samples that underwent WGS and phenotypic AST

Bacterial species	Isolate ID	**ST**	*K. pneumoniae* K locus/O locus	Intrinsic chromosomal ARGs	Phenotypic antibiotic resistance (determined by AST)	Pathogenicity
*Citrobacter* sp.	BNP37	ST1272	–	*bla*GIL-1^*^	AMP, AMOX	Opportunistic pathogen
*C. braakii*	BNP58	ST1271**	–	*bla*CMY-101^‡^	AMP, AMOX, AMC, KZ, CTX	Opportunistic pathogen
*K. oxytoca*	BNP21	ST704	–	*bla*OXY-2–8^‡^	AMP, AMOX, KZ(I)	Opportunistic pathogen
*K. oxytoca*	BNP27	ST703	–	*bla*OXY-2–8^‡^	AMP, AMOX, KZ(I)	Opportunistic pathogen
*K. oxytoca*	BNP40	ST288	–	*bla*OXY-2–8^‡^	AMP, AMOX, KZ(I)	Opportunistic pathogen
*K. oxytoca*	BNP44	ST850**	–	*bla*OXY-2–1	AMP, AMOX, KZ	Opportunistic pathogen
*K. oxytoca*	BNP74	ST179	–	*bla*OXY-2–2	AMP, AMOX, KZ	Opportunistic pathogen
*K. oxytoca*	BNP93	ST287	–	*bla*OXY-2–7	AMP, AMOX, KZ(I)	Opportunistic pathogen
*K. pneumoniae*	BNP96	ST9093**	KL10/O5	*bla*SHV-172^§^	AMP, AMOX	Opportunistic pathogen
*Scandinavium* sp.	BNP24	–	–	*ampC* ^¶^	AMP, AMOX, AMC, KZ	Rare pathogen
*Scandinavium* sp.	BNP63	–	–	*ampC* ^¶^	AMP, AMOX, AMC, KZ	Rare pathogen

Antibiotics: AMC, amoxicillin plus clavulanic acid; AMOX, amoxicillin; AMP, ampicillin; CTX, cefotaxime (third-generation cephalosporin); I, intermediate resistance; KZ, cefazolin (first-generation cephalosporin).

*Closest match (89.3%) – *bla*GIL-1 – *Citrobacter gillenii* (GenBank accession NG_049142).

†Closest match (99.21%) – *bla*CMY-101 – *C. braakii* (GenBank accession OY762811).

‡Closest match (99.43–99.89%) – *bla*OXY-2–8 – *K. oxytoca* (GenBank accession AY055205).

§Closest match (99.88%) – *bla*SHV-172– *K. pneumoniae* (GenBank accession KF513177).

¶Closest match (86.0%) – *ampC* – *S. goeteborgense* (GenBank accession CP054058).

**Novel ST.

The 11 amoxicillin-resistant isolates carried chromosomally located intrinsic beta-lactamase genes, which conferred phenotypic resistance to amoxicillin and ampicillin (Table 2). All six *K. oxytoca* isolates carried *bla*OXY-2 gene variants, with two isolates also exhibiting resistance to cefazolin and the remaining four *K. oxytoca* isolates exhibiting intermediate resistance to cefazolin (Table 2). The *C. braakii* isolate (BNP58) exhibited additional resistance to amoxicillin+clavulanic acid and first- and third-generation cephalosporins (conferred by *bla*CMY-101) (Table 2). Both *Scandinavium* spp. isolates exhibited resistance to amoxicillin+clavulanic acid and cefazolin (a first-generation cephalosporin), which was likely conferred by a novel *ampC* gene that showed 86.0% identity to *ampC* associated with *S. goeteborgense* (GenBank accession CP054058.1) (Table 2). Genome analysis of the 11 isolates determined that none carried any acquired resistance genes, including *bla*TEM, nor exhibited phenotypic resistance to carbapenems, fluoroquinolones, trimethoprim plus sulphonamides, tetracyclines, chloramphenicol or gentamicin, as determined by EUCAST disc diffusion (Table S3).

All six koala *K. oxytoca* isolates carried the virulence factor yersiniabactin, and none of the koala *Klebsiella* isolates carried aerobactin or colibactin. However, we note that the Kleborate database was designed to capture allelic variants among *K. pneumoniae*, and divergent variants among *K. oxytoca* may not be detected. *K. oxytoca* isolate BNP27 (ST703) carried an IncFII plasmid, with the replicon sequence showing 92.3% identity to IncFII(S) and 85.1% identity to IncFII(pAR0022) (GenBank accessions CP000858 and CP024882, respectively). The *Citrobacter* spp. isolate BNP37 (ST1272) carried a Col plasmid, with the replicon sequence showing 85.2% identity to Col(pHAD28) (GenBank accession KU674895).

### Associations between carriage of opportunistic pathogens and koala metadata

The amoxicillin-resistant isolates determined to be opportunistic pathogens (*n*=9) were detected in adult koalas (91/98 koalas), and no opportunistic pathogens were isolated from dependent joeys (7/98 koalas) (Table S1).

The seven koalas carrying *Klebsiella* isolates (*K. oxytoca* or *K. pneumoniae*) had BCSs of ‘poor’ (3/7, 42.9%) or ‘fair’ (4/7, 57.1%), whereas the majority of koalas that did not carry *Klebsiella* had a BCS of ‘fair’ (42/91, 46.2%) or ‘good/excellent’ (35/91, 38.5%) (Table S4). However, the association of *Klebsiella* presence with poorer body condition compared to non-*Klebsiella-*carrying koalas was not statistically significant (Fisher’s exact test *P*-value=0.0907) (Table S4).

Of the seven *Klebsiella* isolates, six were carried by female koalas and one *K. oxytoca* isolate by a male koala; however, this was not significantly different from non-*Klebsiella*-carriers due to unequal sampling of males (*n*=35) and females (*n*=63) (Fisher’s exact test *P*-value=0.4159) (Table S4). The two *Citrobacter* spp. were carried by one male and one female koala (Table S1). None of the seven female koalas carrying amoxicillin-resistant opportunistic pathogens had pouch young or back young (Table S1).

### Phylogenetic analysis of koala *K. oxytoca* and *K. pneumoniae* isolates

**Fig. 1.**
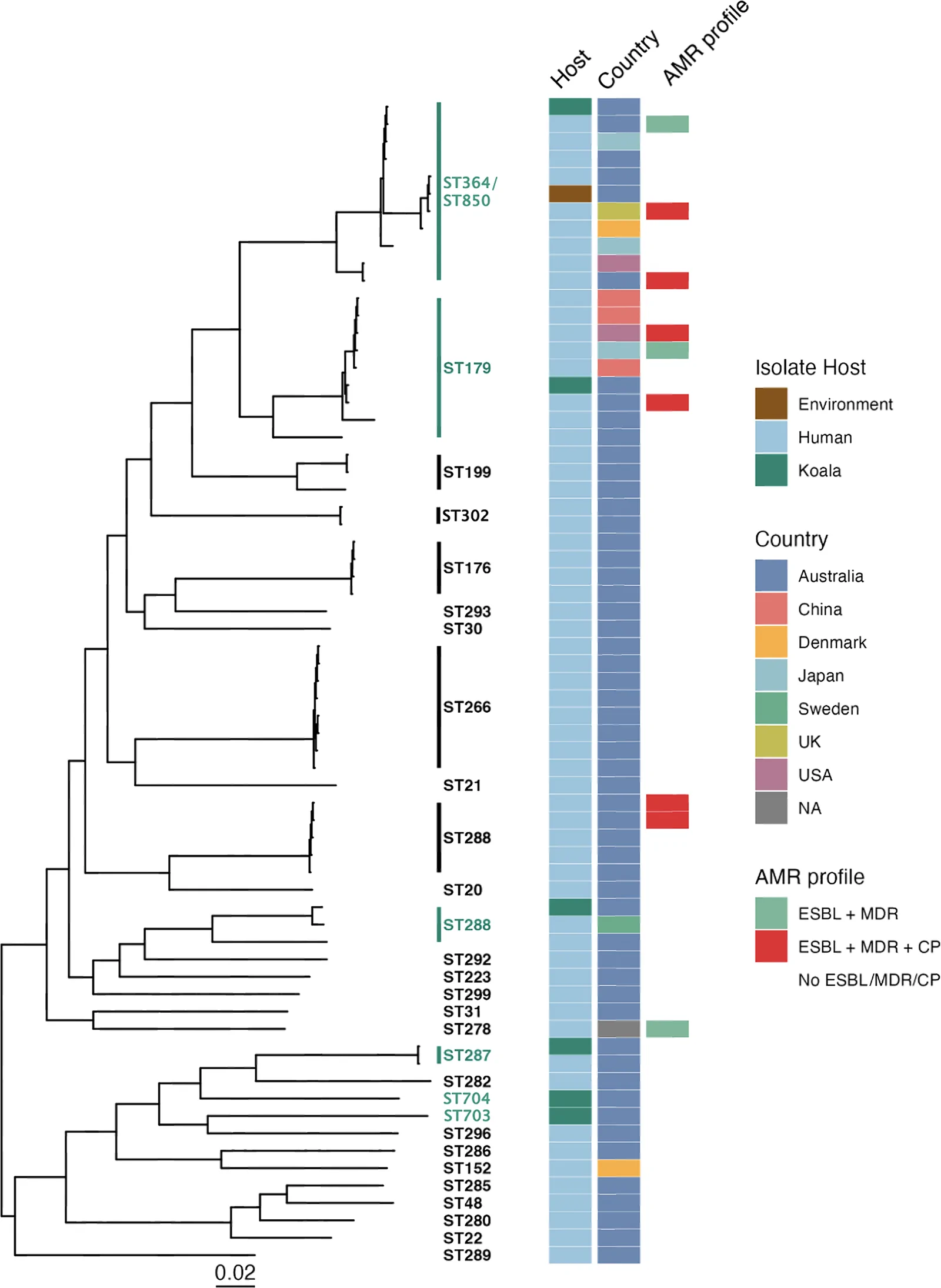
Phylogeny of six *K. oxytoca* isolates from koalas, additional related *K. oxytoca* isolates identified on Pathogenwatch/BIGSdb (available at https://next-docs.pathogen.watch) and Australian-sourced *K. oxytoca* isolates from Stewart *et al*. [54]. Midpoint-rooted core genome maximum-likelihood phylogeny of 70 *K*. *oxytoca* isolates (koala, *n*=6; environmental, *n*=1; and human, *n*=63). Tips are labelled with STs, with STs containing a koala isolate coloured in green and others in black. Heatmap to the right of the tree indicates isolate host, country of isolation and AMR profile, indicating presence or absence of AMR determinants (CP=presence of carbapenemase genes, ESBL=extended spectrum beta-lactamase, MDR=resistant to ≥3 drug classes other than ampicillin/amoxicillin). All *K. oxytoca* isolates carried the virulence factor yersiniabactin (*Ybt*).

Phylogenetic analysis of *K. oxytoca* isolates from koalas (*n*=6) and the sourced *K. oxytoca* collection (*n*=64 isolates) found that four of six koala isolates clustered with strains sourced from humans, including isolates associated with clinical infections (Fig. 1). Koala *K. oxytoca* isolates BNP40 (ST288), BNP44 (ST850), BNP74 (ST179) and BNP93 (ST287) showed 1653, 90, 331 and 94 SNVs with their closest human-sourced isolates, respectively (Table S5). Two koala *K. oxytoca* isolates, BNP21 (ST704) and BNP27 (ST703), were not closely related to any other strains (Fig. 1), showing 25,520 and 27,672 SNVs with their closest human-sourced isolates, respectively (Table S5).

**Fig. 2.**
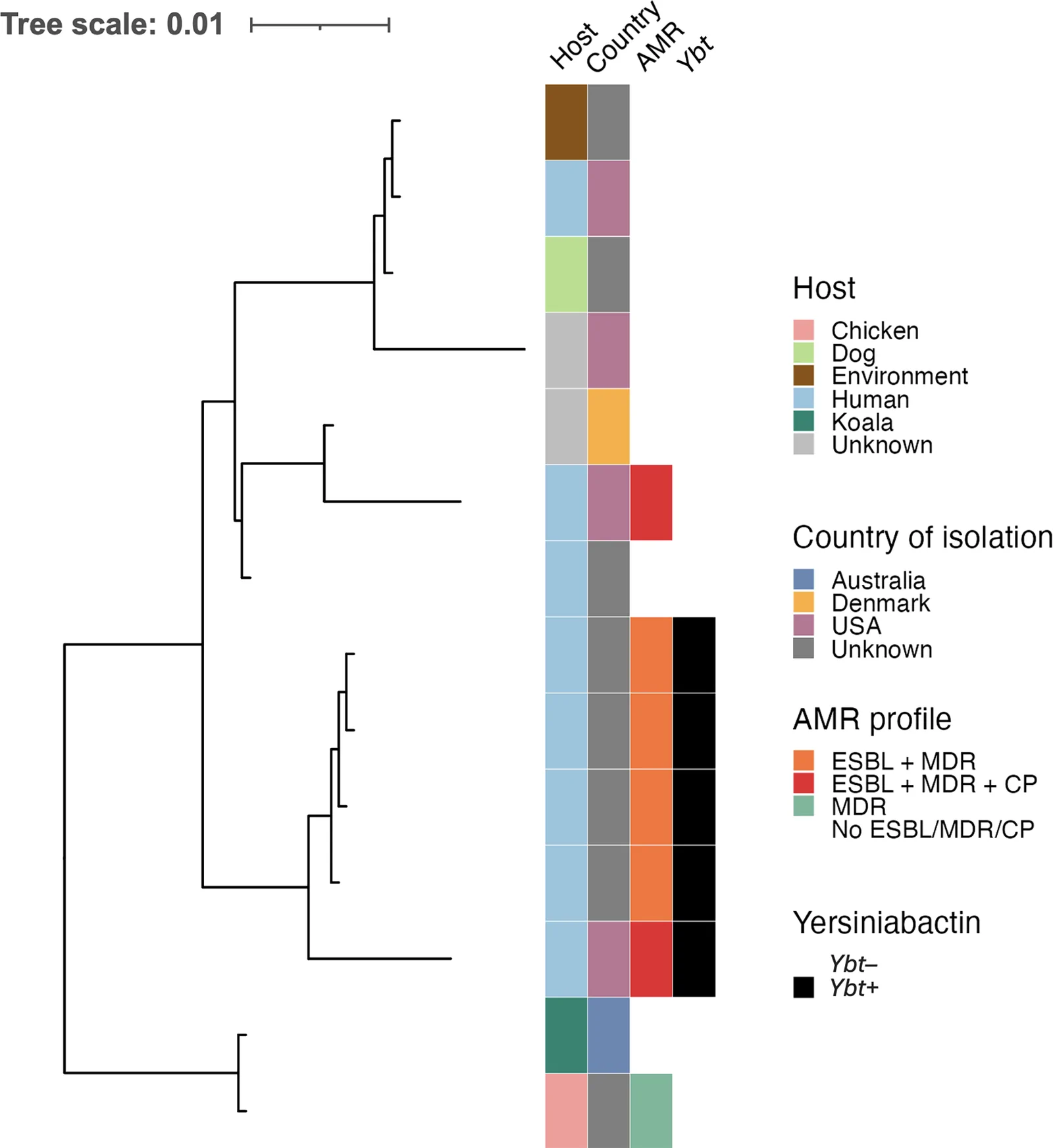
Outgroup-rooted maximum-likelihood phylogeny of 14 *K*. *pneumoniae* LIN Sublineage 1944 (SL1944) isolates (koala, *n*=1; human, *n*=8; domestic animals, *n*=2; environmental, *n*=1 and unknown, *n*=2) identified in the BIGSdb (https://bigsdb.pasteur.fr/cgi-bin/bigsdb/bigsdb.pl?db=pubmlst_klebsiella_isolates). The phylogeny has been outgroup-rooted using a non-SL1944 *K. pneumoniae* isolate (removed for clarity). Heatmap to the right of the tree indicates isolate host, country of isolation, AMR profile, indicating presence or absence of AMR determinants (CP=presence of carbapenemase genes, ESBL=extended spectrum beta-lactamase, MDR=resistant to ≥3 drug classes other than ampicillin/amoxicillin), and presence or absence of virulence determinants Yersiniabactin (*Ybt*) and Aerobactin (*Iuc*).

Phylogenetic analysis of *K. pneumoniae* isolates from a koala (BNP96 and ST9093) and 13 related isolates found that the koala isolate was most closely related to a MDR ST1944 isolate sourced from chicken meat (139 SNVs) (Fig. 2). These two isolates belonged to LIN Sublineage 1944 (SL1944) that also included MDR strains of *K. pneumoniae* associated with human clinical infections (Fig. 2).

### Genetic relationships of koala *Citrobacter* isolates

**Fig. 3.**
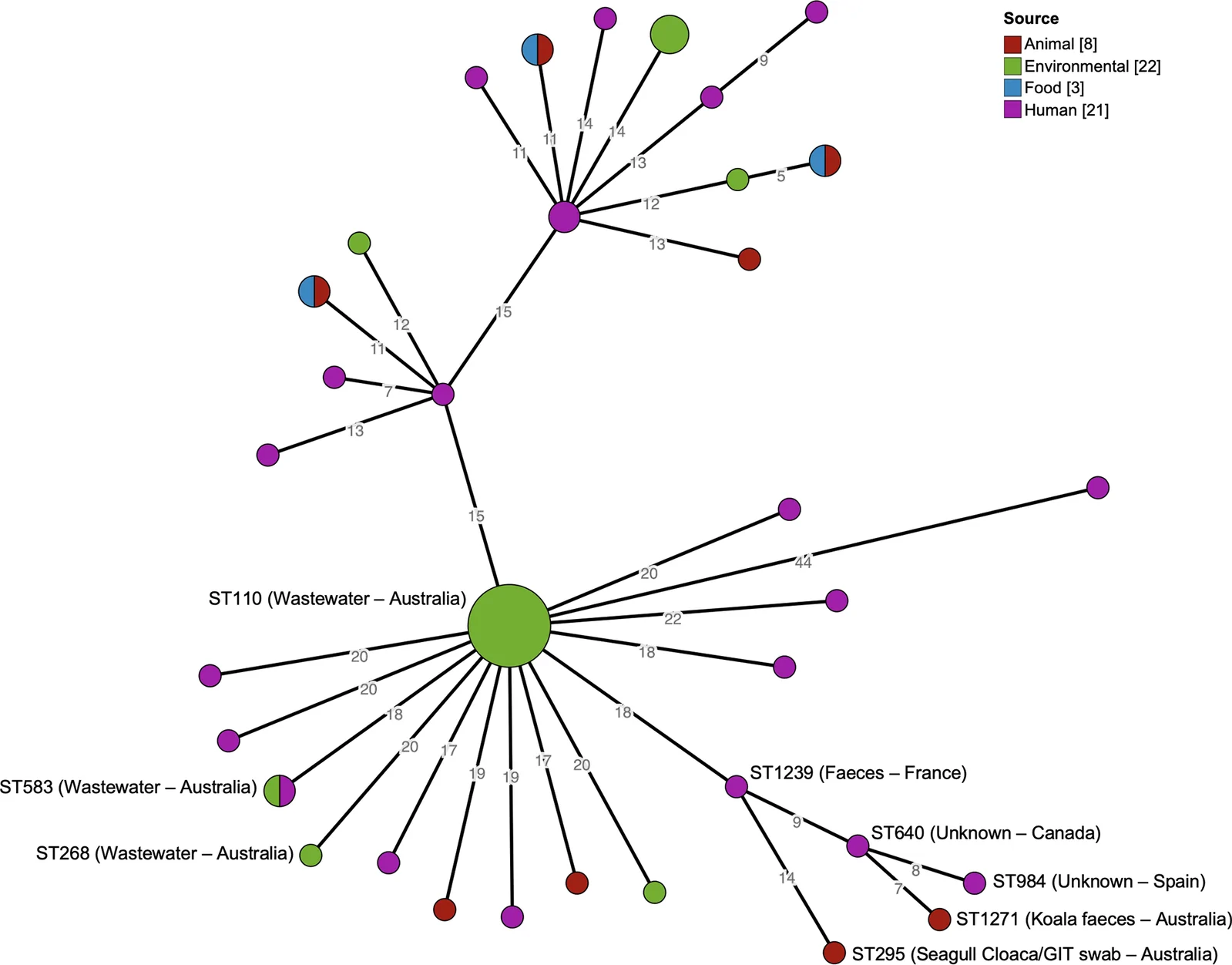
GrapeTree depicting the genetic relationship of the koala *C. braakii* isolate (BNP58, PubMLST ID 1067, ST1271) and other *C. braakii* isolates identified in the PubMLST *Citrobacter* genome collection (*n*=53). The minimum spanning tree was constructed using GrapeTree and MSTree V2 [56] based on the PubMLST *Citrobacter* MLST scheme (includes seven MLST housekeeping loci) and the ribosomal MLST scheme (includes 53 ribosomal loci) [43]. Branch lengths indicate the allelic differences between isolates. The koala *C. braakii* isolate, closely related isolates and other Australian-sourced isolates are labelled with ST, detailed isolate source and country of detection.

Based on GrapeTree analysis of the koala *C. braakii* isolate and 53 *C*. *braakii* isolates in the PubMLST genome collection, the koala isolate was most closely related to three non-Australian human-sourced isolates and one isolate sourced from a seagull in Sydney, Australia (Fig. 3). The koala *C. braakii* isolate showed ≥7 allelic differences to the four most closely related isolates (Fig. 3). Notably, the koala isolate was not closely related to any of the multiple *C. braakii* strains sourced from wastewater in Adelaide, Australia (all showed ≥34 allelic differences to the koala isolate) (Fig. 3).

The koala *Citrobacter* spp. isolate was not closely related to any other *Citrobacter* isolates in the PubMLST genome collection and was most closely related to five *Citrobacter gillenii* isolates sourced from Australian seagulls (*n*=2), human urine (*n*=1) and the environment (*n*=2) (all showed ≥29 allelic differences to the koala isolate) (Fig. S1).

### Phylogenetic analysis of koala *Scandinavium* isolates

Phylogenetic analysis of the two koala *Scandinavium* sp. isolates, assembled *Scandinavium* spp. genomes available in GenBank (*n*=22) and two representative *K. aerogenes* genomes from GenBank placed the two koala isolates in a novel *Scandinavium* lineage (Fig. S2). The koala *Scandinavium* sp. isolates belonged to a clade which also contained *Scandinavium* sp. isolates sourced from Australian soil (*n*=2) and *S. lactucae* isolates sourced from lettuce in South Korea (*n*=4) (Fig. S2). The koala isolate genomes shared 99.4% ANI with each other but<95% ANI with all other genomes (Fig. S3). The most closely related genomes shared ~89.5% ANI (89.4–89.5% ANI with the Korean *S. lactucae* genomes and 86.5% with the *Scandinavium* sp. from Australian soil), suggesting that the koala strains may represent a novel species (Fig. S3).

## Discussion

This study identified intrinsically amoxicillin-resistant bacterial isolates considered to be opportunistic pathogens (*Klebsiella* spp. and *Citrobacter* spp.) in the faecal microbiome of 9 of 98 (9.2%) wild koalas from BNP, South Australia. Two additional amoxicillin-resistant isolates (*Scandinavium* sp.) considered to be rare pathogens were also detected. Nine of the eleven amoxicillin-resistant isolates also exhibited resistance to first-generation cephalosporins, three isolates were also resistant to amoxicillin+clavulanic acid and one isolate also exhibited resistance to third-generation cephalosporins. Beta-lactam resistance was conferred by diverse intrinsic chromosomal genes (*ampC*, *bla*CMY, *bla*GIL, *bla*OXY and *bla*SHV) and no isolates carried acquired resistance genes. The *bla*TEM gene was not identified in any of the 11 isolates, which is consistent with the absence of *bla*TEM detection in faecal DNA samples from the same koalas in a previous study [24].

The 11 WGS koala isolates belonged to four Gram-negative taxa, comprising *K. oxytoca, K. pneumoniae, Citrobacter* spp. and a novel *Scandinavium* species. *K. pneumoniae* and *K. oxytoca* have previously been reported in the faecal microbiome of koalas; however, published literature is sparse. Three publications have reported *K. pneumoniae* isolation from faeces [37, 58, 59], one study also isolated *K. oxytoca* [37] and one study isolated *K. pneumoniae* from koala ‘pap’ – special faeces produced by a female koala for consumption by her joey in order to establish the required adult gut microflora for digesting eucalypt leaves [60]. This study appears to be the first to perform genetic characterization and antibiotic susceptibility testing on *Klebsiella* spp. isolated from koala faeces. A literature search for *Citrobacter* spp. presence in koala faeces returned no results; however, *Citrobacter koseri* has been isolated from a wound in a wild koala [61]. This study on koalas in South Australia appears to be the first to report and characterize *Citrobacter* spp. and *Scandinavium* spp. in koala faeces.

In koalas, *K. pneumoniae* and *K. oxytoca* have been proposed to play a role in the degradation of tannins present in eucalypt leaves (the koala’s principal diet) in the koala gut [62]. However, several studies have concluded that tannin-protein complex-degrading bacteria belonging to the family *Enterobacteriaceae* – which includes *K. pneumoniae* and *K. oxytoca* – are uncommon and present at low abundance in the koala gut microbiome, suggesting that they do not have a major role in diet detoxification [63–65].

*K. pneumoniae* is a common cause of infections in human hospital patients, including pneumonia, urinary tract infections, bacteraemia and sepsis, and is increasingly responsible for community-acquired infections, particularly pneumonia, meningitis and pyogenic liver abscess [66]. Over the past decade, *K. oxytoca* has emerged as a significant opportunistic nosocomial pathogen associated with systemic infections, particularly in neonatal infants [67], and is increasingly associated with community-acquired infections and sepsis [68]. In koalas, *K. pneumoniae* has been isolated from systemic and localized infections, including intestinal tract infections, pharyngitis and pouch infections in female koalas [58, 59, 69], demonstrating their ability as opportunistic pathogens in koalas [59]. Both *K. pneumoniae* and *K. oxytoca* have also been linked to systemic infection, septicaemia and mortality in koala pouch young [59, 69].

Little genetic characterization of *Klebsiella* spp. associated with clinical infections in koalas has been performed. In a study of captive koalas, *K. pneumoniae* ST278 was found to be associated with pouch mortality [69]. *K. pneumoniae* ST278 has also been associated with human blood, intra-abdominal and urinary tract infections [54, 70, 71], and neonatal septicaemia in pigs [72]. In this study, WGS and phylogenetic analysis determined that four koala *K. oxytoca* isolates (ST179, ST287, ST288 and ST850) belonged to lineages that included strains associated with clinical disease in humans, and notably the ST287 and ST850 isolates differed by <100 SNVs compared to the most closely related human derived isolate. *K. oxytoca* ST179 has been associated with an outbreak (pneumonia, sepsis and perforated necrotizing enterocolitis) in a human neonatal intensive care unit [73]. ST287 and ST288 have been reported in adult human pneumonia infections in Australia [54], ST179 and ST287 have been cultured from blood (PubMLST ID 949 and ID 615, respectively) and ST288 has also been associated with sepsis in a human patient [68]. ST364, the closest relative to koala ST850, has been isolated from human bloodstream infections [74] and clinical infections in horses [75]. The detection of *K. oxytoca* and *K. pneumoniae* isolates in female koalas (all without pouch or back young) is concerning, especially given the association of *K. oxytoca* and *K. pneumoniae* with mortality in pouch young [59, 69].

*Citrobacter* spp., particularly *C. freundii*, *C. koseri* and *C. braakii* are increasingly associated with opportunistic infections in humans, especially neonatal infections and in patients with underlying conditions and/or are immunocompromized [76, 77]. A literature search identified multiple reports of opportunistic infections with *C. braakii*, including urinary tract infection, pneumonia, bacteraemia plus sepsis and diarrhoea [77–80]. The single published report of *C. koseri* infection in a koala was associated with a nailbed infection in a wild koala, which cultured mixed growth of *C. koseri*, *E. cloacae* and a *Lonepinella*-like organism [61]. The nailbed wound infection failed to respond to amoxicillin administration, but partial improvement was seen following chloramphenicol administration; however, the affected claw required surgical removal [61]. The koala *C. braakii* isolate identified in this study exhibited phenotypic resistance to ampicillin, amoxicillin±clavulanic acid, cefazolin (first-generation cephalosporin) and cefotaxime (third-generation cephalosporin) due to the intrinsic *bla*CMY-101 gene. Thus, *C. braakii* should be considered an opportunistic pathogen in koalas, especially in individuals with underlying conditions such as chlamydial disease, koala retrovirus disease or antibiotic-induced gut dysbiosis [31, 81]. The second koala *Citrobacter* sp. isolate was clustered with *C. gillenii* isolates, which have been reported to cause disease outbreaks in fish [82, 83] and human urinary tract infection (PubMLST ID 528).

The significance of finding a putative novel *Scandinavium* species in koalas is unclear. The genus *Scandinavium* was first described in 2019, when a novel isolate, designated *S. goeteborgense*, was isolated from a wound infection of an adult human patient in Sweden; thus, *S. goeteborgense* is considered to be an opportunistic pathogen [84]. Since 2019, numerous other *Scandinavium* spp. have been identified, with most detected in environmental sources, and it is currently unknown if these *Scandinavium* spp. are also opportunistic pathogens [85, 86]. The koala *Scandinavium* sp. isolates carry an *ampC* gene that showed high similarity to the *ampC* gene identified *S. goeteborgense* isolate CCUG 66741 (GenBank accession CP054058), but they did not carry the novel fluoroquinolone resistance gene *qnrB96* also identified in *S. goeteborgense* isolate CCUG 66741 [84]. Given that the novel koala *Scandinavium* sp. exhibited phenotypic resistance to ampicillin, amoxicillin, amoxicillin plus clavulanic acid and cefazolin (first-generation cephalosporin), it would be prudent to consider it a potential pathogen in koalas.

The colonization of koala gut microbiomes by clinically important opportunistic pathogenic bacteria that exhibit intrinsic resistance to beta-lactam antibiotics, including amoxicillin, amoxicillin+clavulanic acid and cephalosporins, identifies an important disease risk factor [30]. Of particular concern is the detection of *Klebsiella* strains belonging to lineages associated with clinical infections in humans, given the association of *K. pneumoniae* and *K. oxytoca* with opportunistic infections [59] and the loss of pouch young in koalas [59, 69]. Furthermore, administration of penicillins or cephalosporins to treat bacterial infections may select for these resistant pathogens and exacerbate or facilitate opportunistic infections [29, 30].

For koalas, the risk of developing antibiotic-induced gastrointestinal dysbiosis [31] may also be exacerbated by the presence of amoxicillin- and cephalosporin-resistant opportunistic pathogens including *K. pneumoniae* and *K. oxytoca* [59]. Although *K. pneumoniae* and *K. oxytoca* may be present at low abundance in healthy koala gut microbiomes [63, 64], increased abundance in response to amoxicillin administration may contribute to gut dysbiosis [87, 88]. Given that high amoxicillin administration was reported in fire-affected koalas in 2019/2020 (26.5% of admitted koalas) [28], the detection of intrinsically amoxicillin-resistant *K. pneumoniae* and *K. oxytoca* in wild koalas raises significant concerns. This study highlights the importance of good antimicrobial stewardship when treating wildlife [89] and reinforces the need for judicious antibiotic administration, especially in species like koalas [28], which have inherently sensitive gut microbiomes [31].

The presence of opportunistic bacterial pathogens in koala microbiomes, particularly strains associated with human clinical infections, is yet another threat to the conservation of this endangered species. The observed intertwined microbial ecology between humans, domestic animals, the environment and wildlife highlights the significant One Health issue of microbial pathogens [90]. Additionally, anthropogenic-driven threats (heat waves, drought, habitat loss, urbanization, etc.) [91, 92] are increasingly resulting in hospital admissions of injured or sick koalas [27, 28, 93]. The combination of opportunistic bacterial pathogen carriage and increasing antibiotic administration [28] poses an emerging disease risk for Australia’s koalas, even in the absence of acquired antibiotic resistance. This study highlights the need for further research on the impacts of these opportunistic bacterial pathogens on koala health and for antimicrobial stewardship and microbiome disturbance to be integrated into koala conservation management strategies.

## Abbreviations

AMR: antimicrobial resistance
ANI: Average Nucleotide Identity
ARG: antimicrobial resistance genes
AST: antimicrobial susceptibility testing
BCS: body condition score
BIGSdb: Bacterial Isolate Genome Sequence Database
BNP: Belair National Park
CLSI: Clinical and Laboratory Standards Institute
EUCAST: European Committee on Antimicrobial Susceptibility Testing
LIN: Life Identification Number
MDR: multidrug resistance
rMLST: ribosomal multilocus sequence typing
SNV: single nucleotide variant
SRA: Sequence Read Archive
ST: sequence type
WGS: whole-genome sequencing
